# Granuloma annulare after starting semaglutide

**DOI:** 10.1016/j.jdcr.2025.05.005

**Published:** 2025-05-30

**Authors:** Tajena Jones, Bridget Myers, Thomas Konia, Danielle M. Tartar, Jonathan Rick

**Affiliations:** Division of Dermatology, Department of Medicine, University of California Davis, Sacramento, California

**Keywords:** annular lesions, GLP-1 agonists, glucagon-like peptide-1, granuloma annulare, granulomatous infiltrate, semaglutide, type II diabetes mellitus

## Introduction

Semaglutide is a weekly injected glucagon-like peptide-1 agonist medication used to treat type 2 diabetes mellitus by increasing insulin levels, delaying gastric emptying, and ultimately inducing weight loss.^1^ In recent years, the use of glucagon-like peptide-1 agonists has increased, with an almost six-fold growth in certain populations.^2^ Potential side effects of semaglutide include severe gastrointestinal adverse reactions, hypoglycemia, and thyroid cancer.^1^ Various case studies published in the Journal of the American Academy of Dermatology have noted cutaneous reactions after starting semaglutide, including exanthematous pustulosis sine pustules, erythema nodosum, and bullous pemphigoid.^3^, ^4^, ^5^ The full side-effect profile remains unknown and given the rapid increase in the popularity of glucagon-like peptide-1 agonists, it is important to provide details of encountered reactions. We report here an eruption of granuloma annulare following the initiation of semaglutide for diabetes management.

## Case presentation

**Fig 1:**
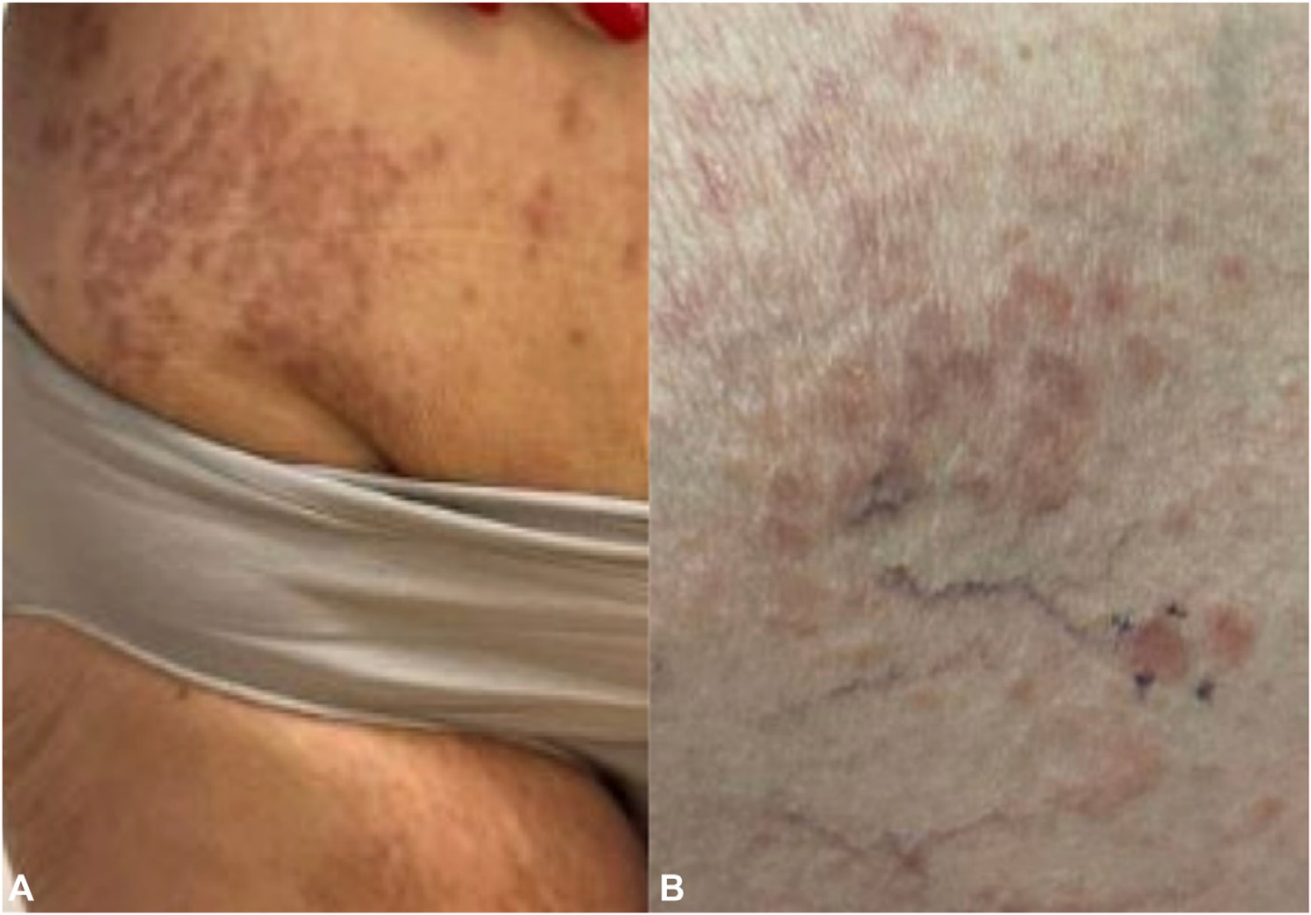
**A,** Nonpruritic annular lesions in with some purpuric color changes on right groin and abdomen. **B,** Focused view of nonpruritic circular lesions in with some purpuric color changes on medial right leg.

**Fig 2:**
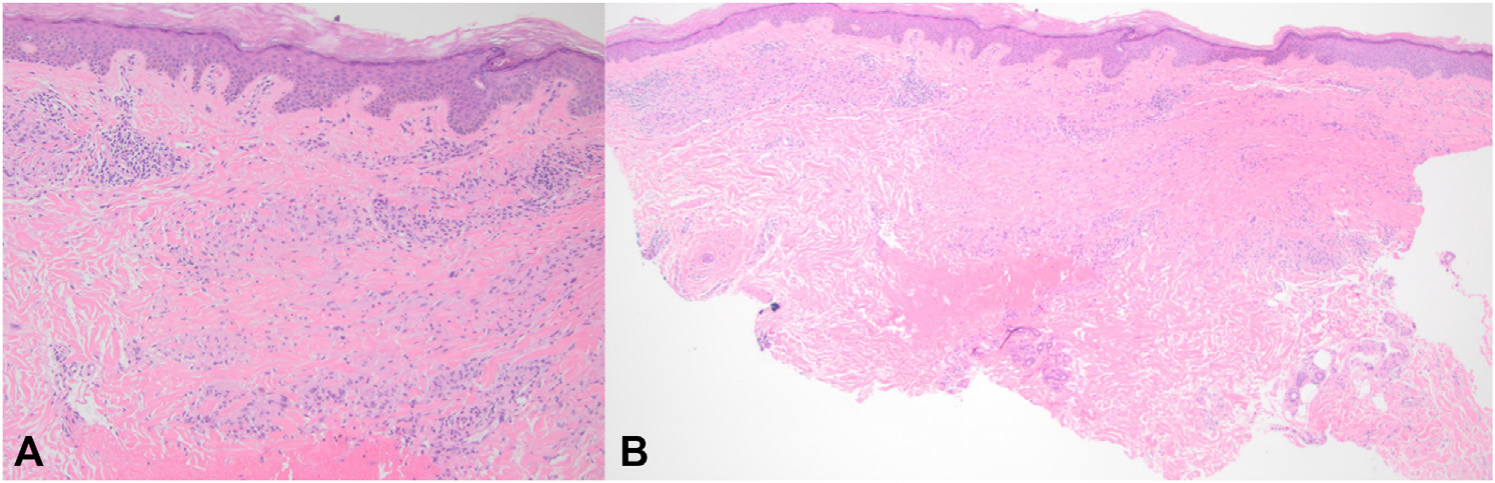
**A,** Punch biopsy of the patient’s right medial leg (200× magnification). **B,** Punch biopsy of the patient’s right medial leg (100× magnification).

A 73-year-old woman with a past medical history of type 2 diabetes mellitus, obesity, hyperlipidemia, lichen planus, and atopic dermatitis presented with a new rash after starting semaglutide. The patient had been started on 0.25 mg of semaglutide and titrated up to 1.0 mg weekly (as per manufacture protocol). Two weeks after increasing her dosage to 1.0 mg, the patient reported an asymptomatic rash and was seen by a primary care provider. Clinical examination revealed an eruption of numerous small erythematous annular plaques involving the hips, lower abdomen, medial thighs, and left wrist. Interestingly, the rash began around her waist, near the site of semaglutide injections. The provider prescribed the patient a 16-day prednisone taper with some improvement, but the rash did not resolve completely. The patient expressed a desire to continue semaglutide and the dose was increased to 2.0 mg weekly. After 2 months on this new dose, the eruption spread to involve her arms and legs, and the patient was referred to dermatology. On dermatologic exam, she was noted to have annular, erythematous patches and plaques that involve her groin (Fig 1, *A*), abdomen (Fig 1, *A*), arms, and legs (Fig 1, *B*). She denied pain, pruritus, and a history of a similar rash. A punch biopsy of the patient’s right medial leg was obtained which revealed a palisaded infiltrate of histiocytes and lymphocytes in the dermis surrounding paucicellular zones containing altered collagen fibers and increased mucin (Fig 2, *A* and *B*). Considering the clinical findings, time course, and biopsy results, the patient was diagnosed with granuloma annulare attributed to semaglutide therapy. The patient elected to continue with semaglutide treatment and began tirzepatide for treatment of granuloma annulare.

## Discussion

Granuloma annulare is a condition characterized by the presence of a granulomatous infiltrate in a ring-shaped, or annular, pattern. The lesions seen in granuloma annulare are typically located on the hands and/or feet, but some variations have a more disseminated distribution.^6^ The etiology of granuloma annulare is uncertain, however, it is associated with conditions such as diabetes mellitus, hyperlipidemia, malignancy, rheumatoid arthritis, and certain infections (ie EBV, Borrelia).^6^

There are many treatment options for granuloma annulare, with the first line being topical and intralesional corticosteroids.^6^ If granuloma annulare persists after corticosteroid therapy, other options include dapsone, hydroxychloroquine, methotrexate, pentoxifylline, sulphasalazine, phototherapy, and targeted immunomodulators.^6^ Our patient was transitioned to tirzepatide and 3 weeks later the lesions faded in intensity. Due to cosmetic concerns expressed by the patient, she was also started in PUVA (Psoralen with Ultra-violet A phototherapy). Ultimately, granuloma annulare is self-limited and resolves within a few years.^7^ Similarly to this patient’s case, there are other cases of granulomatous drug eruptions which have resolve after the discontinuation of the associated drug.^8^ That we know of, we are the first to report a case of disseminated granuloma annulare in response to semaglutide therapy. Physicians should be cognizant of new cutaneous reactions developing after starting semaglutide and continue to report these cases to better understand the risks of the medication and what treatments are effective.
